# Post-COVID-19 Syndrome: A Novel Diagnosis

**DOI:** 10.7759/cureus.28266

**Published:** 2022-08-22

**Authors:** Rishi Kalia, Ravi Kalia, Joshua Musih, Merly Cubelo, Jesal Popat

**Affiliations:** 1 Medicine, Dr. Kiran C. Patel College of Osteopathic Medicine, Nova Southeastern University, Fort Lauderdale, USA; 2 Interventional Cardiology, Florida Medical Clinic, Tampa, USA

**Keywords:** post covid fatigue, post-acute sequelae of covid-19, post-acute covid-19 syndrome, corona virus disease 2019, whole foods plant based diet, autonomic nervous system dysfunction, postural tachycardia syndrome, long-covid-19, covid-19, post-covid-19 complications

## Abstract

Patients with post-COVID-19 syndrome have reported a wide array of symptoms that include autonomic dysfunction. It is hypothesized that this may be secondary to interruption of baroreflex pathways in the carotid arteries or nucleus tractus solitarius, however, confirming studies have yet to be performed. A limited number of studies have highlighted the presence of an exaggerated baroreflex response in patients with a post-COVID-19 syndrome that mirror other chronic autonomic dysfunction-related conditions.

## Introduction

Coronavirus disease 2019 (COVID-19), is caused by the novel coronavirus SARS-CoV-2. Even though most of the infected patients present with mild symptoms or are asymptomatic, around 5% to 8% develop acute complications such as hypoxia and reduced lung compliance requiring non-invasive ventilation or mechanical ventilatory support [1].

As the nature of the COVID-19 global pandemic has evolved, management of COVID-19 has shifted from acute care to management of long-term sequelae. Many physicians have reported patients presenting weeks or months after their initial diagnosis, with non-specific symptoms including fatigue, dyspnea, headache, chest and joint pain, and even orthostatic changes. The awareness of these symptoms has led to the creation of the novel diagnosis post-COVID-19 syndrome (PCS) [2].

Although there are currently no established diagnostic criteria for PCS, the most commonly used criteria in the literature is COVID-19 symptoms persisting for more than 12 weeks from initial symptom onset [3]. To date, limited literature exists regarding the exact pathophysiology of this presentation. One hypothesis is the interruption of baroreflex pathways in the carotid arteries or nucleus tractus solitarius, however, confirming studies have yet to be performed [4]. Multiple studies have highlighted a similar exaggerated baroreflex response in those patients suffering from postural orthostatic tachycardia syndrome (POTS) [5]. Several studies have attempted to utilize treatments for the management of POTS and systemic exertion intolerance disease (SEID) while therapeutic research continues [6].

## Case presentation

A 49-year-old female with a past medical history of persistent headaches and COVID-19 pneumonia (diagnosed in September 2021) presented to the hospital in December 2021 for evaluation of multiple syncopal events. Blood pressure measurements upon admission were 110/76 seated, 98/76 standing, and 111/67 supine; oxygen saturation of 99% and temperature of 98.2 F. Cardiac workup including troponin T was negative and no arrhythmias were found on telemetry. The neurological evaluation included CT, lumbar puncture, and EEG, which were also negative for any pathology. An echocardiogram showed normal systolic function and no wall motion changes (Table 1). The patient reported no history of sudden cardiac death, structural heart disease, valvular heart disease, or premature coronary artery disease.

**Table 1:** Patient lab and test results ASCVD*: atherosclerotic cardiovascular disease

Diagnostic Test	Result	Reference
BP (seated, standing, supine)	110/76 mmHg; 98/76 mmHg; 111/67 mmHg	SBP decreased ≥ 20 when standing and/or DBP decreased ≥ 10 when standing
Calcium Scoring	0	0: No ASCVD* 1-10: <10 % chance of ASCVD 11-100: mild risk of ASCVD 10 -400: moderate to high risk of ASCVD >400: >90% chance of ASCVD [7]
Cardiac Monitoring (3 weeks)	No events	
Troponin T	Negative	<14 ng/L
CT	Negative	
Lumbar Puncture	Negative	
EEG	Negative	
Nuclear Stress Test	No inducing ischemia or prior findings	

Several months after hospitalization, she presented to the cardiology clinic due to ongoing symptoms of tachycardia, headaches, and near syncopal events. A full cardiovascular workup was ordered. Calcium scoring revealed a score of zero. Tilt table testing was negative. The patient was placed on a cardiac monitor for three weeks to detect arrhythmias and no significant ventricular or atrial arrhythmias were identified. A nuclear stress test was performed and showed no inducing ischemia or prior infarct.

The patient was discharged from the hospital on topiramate 50mg and midodrine 5mg which were later discontinued on the initial visit to the cardiology clinic. At the two-month follow-up, the patient was prescribed metoprolol 25mg and instructed to titrate up to 50mg for symptomatic relief of tachycardia, diaphoresis, headaches, and near syncopal events. Additionally, she was suggested to try a whole-foods plant-based diet which she agreed to. The patient reported well-controlled blood pressures and no loss of consciousness during this time period.

Subsequently, on a three-month follow-up, the patient reported complete resolution of her symptoms including chest pain, shortness of breath, lightheadedness, and dizziness. In addition, the patient reported that she had continued following a whole-foods plant-based diet. Metoprolol 50mg was discontinued and an 11-month follow-up was scheduled. The patient was advised to return sooner if any previous symptoms reappeared.

## Discussion

An increasing amount of cardiovascular and neurological events have been identified following recovery from acute COVID-19 infection. Many cases in the medical literature now acknowledge PCS as a possible cause of these associations. One prospective cohort study of 1,733 patients estimated that up to 76% who had COVID-19 reported at least 1 persistent symptom at six-month follow-up [8].

In this patient, the lack of diagnostic workup pointing towards another diagnosis leaves post-COVID-19 syndrome as a tentative diagnosis. Several studies have focused on dysautonomia as an underlying mechanism in PCS patients who present with orthostatic changes. Possible factors include hypovolemia, neurotropism, and inflammation which occur secondary to the initial COVID-19 infection.

Currently, the management of POTS focuses on mostly conservative treatment options. These include increased fluid and salt intake, physical therapy, and avoidance of triggers, amongst other non-pharmaceutical options. In terms of pharmaceutical management for POTS, drugs that increase blood volume, counter tachycardia, or increase vasoconstriction have been experimented with [9].

In this patient, the effectiveness of metoprolol in resolving her symptoms suggests sympathetic dysregulation as an underlying etiology of her symptoms. Although it is possible that her symptoms resolved over time, her lack of improvement at first follow-up post hospitalization and studies showing a long-term continuation of symptoms suggests that her autonomic dysfunction was persistent. Further studies should be conducted comparing treatment vs placebo in PCS patients with autonomic symptoms in order to better determine the efficacy of pharmacological treatment. In addition to her pharmaceutical intervention, this patient also agreed to switch to a whole-foods plant-based diet. Although no current studies exist regarding whole-foods plant-based diets and autonomic regulation, future research into whole-foods plant-based nutrition may be beneficial for those with PCS.

## Conclusions

The purpose of this case report is to add to the limited number of studies regarding the management of the post-COVID-19 syndrome. It establishes a precedent for the role that a whole-foods plant-based diet may play in the recovery of patients with PCS; however, its specific benefits are yet to be established.

Patients with PCS have reported a large variety of symptoms that range from fatigue and dyspnea to orthostatic changes. For patients with sympathetic dysfunction, early recognition and treatment are important to improve their quality of life. As awareness and prevalence of PCS increases, future studies may explore other treatment options which may help to provide patients with relief. Additionally, future research into the precise pathophysiology of PCS may provide valuable insight into this condition and future treatments.

## References

[1] Li X, Ma X (2020). Acute respiratory failure in COVID-19: is it "typical" ARDS?. Crit Care.

[2] Shah W, Hillman T, Playford ED, Hishmeh L (2021). Managing the long term effects of covid-19: summary of NICE, SIGN, and RCGP rapid guideline. BMJ.

[3] Dennis A, Wamil M, Alberts J (2021). Multiorgan impairment in low-risk individuals with post-COVID-19 syndrome: a prospective, community-based study. BMJ Open.

[4] Lu Y, Li X, Geng D (2020). Cerebral micro-structural changes in COVID-19 patients - an MRI-based 3-month follow-up study. EClinicalMedicine.

[5] Chadda KR, Blakey EE, Huang CL, Jeevaratnam K (2022). Long COVID-19 and postural orthostatic tachycardia syndrome- is dysautonomia to be blamed?. Front Cardiovasc Med.

[6] Yong SJ (2021). Long COVID or post-COVID-19 syndrome: putative pathophysiology, risk factors, and treatments. Infect Dis (Lond).

[7] University of Maryland Medical Center (2022). Cardiac Calcium Scoring (Heart Scan). https://www.umms.org/ummc/health-services/imaging/diagnostic/cardiac-calcium-scoring.

[8] Huang C, Huang L, Wang Y (2021). 6-month consequences of COVID-19 in patients discharged from hospital: a cohort study. Lancet.

[9] Kavi L, Gammage MD, Grubb BP, Karabin BL (2012). Postural tachycardia syndrome: multiple symptoms, but easily missed. Br J Gen Pract.

